# Role of STAT3 Expression in Thyroid Cancer: A Meta-Analysis and Systematic Review Based on the Chinese Population

**DOI:** 10.1155/2022/1116535

**Published:** 2022-04-15

**Authors:** Ya-Nan Liang, Zuwang Zhang, Ji Song, Fuwei Yang, Pan Yang

**Affiliations:** ^1^Department of Otolaryngology, University-Town Hospital of Chongqing Medical University, Chongqing 401331, China; ^2^Department of Respiratory, University-Town Hospital of Chongqing Medical University, Chongqing 401331, China; ^3^Department of Emergency, The Second Affiliated Hospital of Chongqing Medical University, Chongqing 400010, China

## Abstract

**Background:**

Signal transduction and activator of transcription 3 (STAT3) is an oncogene with transcriptional activity. In recent years, there have been several studies concerning the clinicopathological significance of the expression of the STAT3 protein in thyroid cancer. However, the results are still inconsistent. In this study, we conducted a meta-analysis to evaluate the relationship between the expression of STAT3 protein and thyroid cancer susceptibility and its clinicopathological characteristics.

**Methods:**

We searched the China National Knowledge Infrastructure (CNKI) database, Chinese Biomedical Literature Database (CBM), Chinese Scientific and Journal Database (VIP), Wanfang, PubMed, and EMBASE. The time frame of the publication search was from the establishment of each of the databases until December 2021. We performed a meta-analysis to quantitatively evaluate the relationship between the expression of the STAT3 protein in thyroid cancer and its clinicopathological characteristics.

**Results:**

A total of eight articles were included in the meta-analysis, covering 448 thyroid cancer patients and 227 controls. Results indicated that the expression of STAT3 protein in thyroid cancer tissue is highly expressed (OR = 14.41, 95% CI (6.94, 29.91), *p* < 0.001). Besides, we also discovered that STAT3 protein is negatively correlated with thyroid cancer tumor diameter and TNM stage (OR = 0.13, 95% CI (0.05, 0.33), *p* < 0.001; OR = 0.40, 95% CI (0.24, 0.67), *p* < 0.001) and positively correlated with lymph node metastasis (OR = 2.83, 95% CI (1.08, 7.46), *p* = 0.035). However, STAT3 expression is not related to gender (OR = 0.88, 95% CI (0.54, 1.44), *p* = 0.609), age (OR = 0.54, 95% CI (0.21, 1.36), *p* = 0.191), capsular invasion (OR = 2.98, 95% CI (0.23, 38.29), *p* = 0.403), or tumor multiplicity (OR = 0.25, 95% CI (0.003, 19.28), *p* = 0.533).

**Conclusions:**

This study reveals that STAT3 protein expression is significantly related to the susceptibility and clinicopathological characteristics of thyroid cancer. It also suggests that STAT3 may be a potential predictor of the clinical progression of thyroid cancer.

## 1. Introduction

Thyroid cancer is the most common tumor in the endocrine system, and its incidence rate is gradually increasing [1, 2], yet its pathogenesis is still not clear. Approximately 5–10% of clinically discovered thyroid nodules are thyroid cancer [3]. At present, thyroid cancer is usually clinically diagnosed by thyroid biopsy. However, the lack of early laboratory biochemical indicators has caused certain difficulties in the prevention, timely treatment, and prognosis of its early lymph node metastasis. Therefore, further exploration of markers related to the progression and metastasis of thyroid cancer is of great significance for improving the survival rate of thyroid cancer and guiding its treatment.

Previous studies have located many biomarkers of thyroid cancer, including circulating microRNAs, forkhead box Q1 (FOXQ1), and p53 [4–7]. These biomarkers are all related to the clinicopathology and prognosis of thyroid cancer. Current prognostic indicators include tumor size, location, stage, recurrence rate, and metastasis, but the measurement of these indicators is inaccurate and ineffective. Therefore, it is necessary to find a representative biomarker that can provide a reliable and effective prognosis.

Signal transduction and activator of transcription (STAT) is a family of proteins that regulate cell proliferation, growth, and differentiation and are involved in the signal transduction pathways of various cytokines and growth factors in the body [8, 9]. The STAT family has seven members, of which STAT3 is one of its prominent elements. STAT3 controls many physiological processes including proliferation, differentiation, survival, development, and inflammation. Besides, it is abnormally expressed under certain pathological conditions such as human tumors [10–12]. STAT3 phosphorylates into p-STAT3 under the action of Janus kinase (JAKS), enters the nucleus and binds to specific DNA sequences, regulates the transcription and expression of related target genes, promotes tumor cell proliferation and drug resistance, and inhibits tumor cell apoptosis [12]. Studies have shown that the expression of STAT3 protein is related to poor prognosis of lung cancer [13], breast cancer [14], liver cancer [15], colorectal cancer [16], and prostate cancer [17]. Regarding the relationship between STAT3 and thyroid cancer, existing research has failed to reach a consensus. In this study, meta-analysis was used to clarify the relationship between STAT3 expression and clinicopathological factors in thyroid cancer.

## 2. Materials and Methods

We conducted this review following the Preferred Reporting Items for Systematic Reviews and Meta-Analyses (PRISMA) guidelines. Moreover, we have registered this systematic review and meta-analysis in the Open Science Framework (Registration number: DOI 10.17605/OSF.IO/S5DK6).

### 2.1. Literature Search

Two researchers independently searched the following databases for systematic retrieval: China National Knowledge Infrastructure (CNKI), Chinese Biomedical Literature Database (CBM), Chinese Scientific and Journal Database (VIP), Wanfang database, PubMed, and EMBASE. The search period was from the formation of the databases to December 2021. The search keywords were as follows: STAT3 transcription factor, signal transducer and activator of transcription protein 3, STAT3 protein, thyroid neoplasms, thyroid cancer ^*∗*^, thyroid carcinoma ^*∗*^, thyroid adenoma ^*∗*^, and thyroid tumor ^*∗*^.

### 2.2. Selection Criteria

Study inclusion criteria included the following: cohort studies or case-control studies involving STAT3 and thyroid cancer, and the language was limited to Chinese and English; the case group included specimens of patients with postoperative pathological diagnosis of thyroid cancer, while the control group included pathological diagnoses of normal thyroid cancer tissue in or adjacent to the tumor; and detection of expression levels of the STAT3 protein in tissue samples by immunohistochemistry. Exclusion criteria included the following: abstracts, case reports, summaries, and meeting minutes and repeated publications or repeated data.

### 2.3. Data Extraction

Two researchers independently screened the titles, abstracts, and full texts of the selected articles. In case of disagreements during data extraction, the researchers managed to reach a consensus through discussion. If no agreement could be reached, a third researcher made the final decision. The extracted content mainly included first author, publication time, age, gender, country, language, sample size of the case group and the control group, lymph node metastasis, TNM staging, and research type.

### 2.4. Quality Assessment

We employed the Newcastle–Ottawa scale (NOS) to evaluate the quality of the included literature. The focus of the evaluation mainly included the selection and determination of the research object, whether the groups were comparable, and the measurement of exposure factors. The highest score on the scale is 9 points, and studies with scores greater than 6 are considered high-quality articles [13–15]. Two researchers independently calculated the NOS scores, and they achieved consistency.

### 2.5. Statistical Analysis

For this meta-analysis, we utilized Stata 14.0 software. Moreover, we applied odds ratios (OR) and 95% confidence intervals (CIs) to evaluate the impact of STAT3 expression on clinicopathological characteristics. Also, we analyzed the heterogeneity of the included studies using the *P* value and *I*^2^. In the results, if *P* < 0.1, *I*^2^ > 50%, and there is a certain statistical heterogeneity between the studies, then we performed the meta-analysis using a random model; however, if *P* > 0.1, *I*^2^ < 50%, and the possibility of heterogeneity between the studies was small, then we used a fixed model for the meta-analysis. Finally, we conducted a sensitivity analysis on the included studies. We used Egger's test and Bagger's test to determine the possibility of publication bias. Here, *P* < 0.05 indicated that the difference was statistically significant.

## 3. Results

### 3.1. Literature Search Results

**Figure 1:**
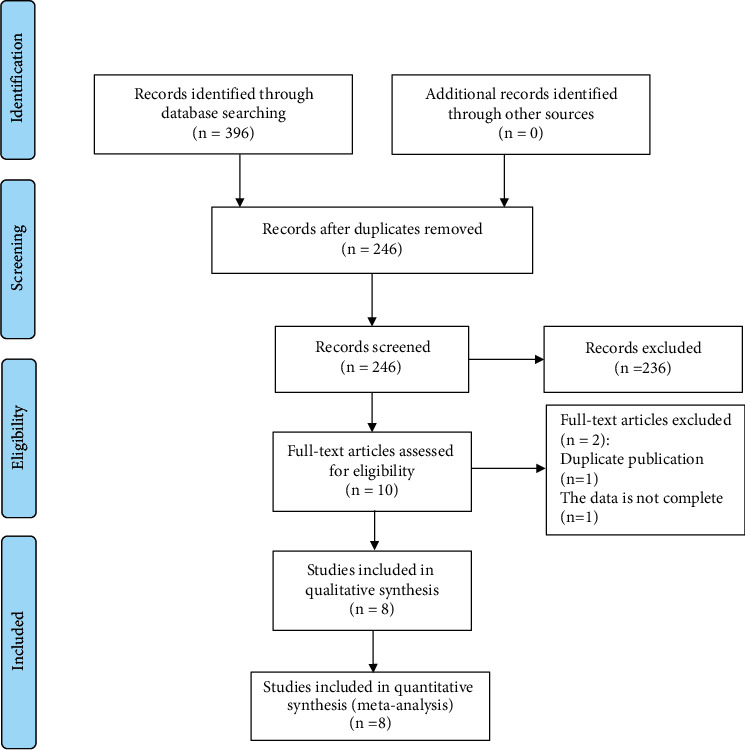
Flow diagram showing the literature filtration process.

The eight included articles [25–32] were published between 2010 and 2017 in China, and all were case-control studies.  Figure 1 shows a flowchart of the literature screening process. All eight articles reported on the expression of STAT3 in thyroid cancer tissue and normal thyroid tissue. Six reports described the relationship between STAT3 expression in thyroid cancer tissue and lymph node metastasis, while three reported the relationship between STAT3 expression in thyroid cancer tissue and tumor TNM staging. Also, four articles explained the relationship between the expression of STAT3 in thyroid cancer tissue and age and gender.  Table 1 provides specific information from the included literature.

### 3.2. Association between STAT3 Expression and the Clinicopathological Parameters of Thyroid Cancer

**Figure 2:**
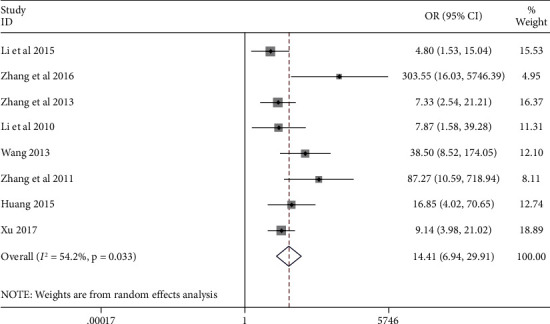
Forest plot showing the relationship between STAT3 protein expression and thyroid cancer.

The eight articles [16–23] contained data from 448 thyroid cancer patients and 227 healthy controls. Results suggested that compared with normal tissue, the expression of STAT3 in thyroid cancer tissue is highly expressed (Figure 2) (OR = 14.41, 95% CI (6.94, 29.91), *p* < 0.001). Besides, we also found that STAT3 is negatively correlated with thyroid cancer tumor diameter and TNM stage (OR = 0.13, 95% CI (0.05, 0.33), *p* < 0.001; OR = 0.40, 95% CI (0.24, 0.67), *p* < 0.001), but positively correlated with lymph node metastasis (OR = 2.83, 95% CI (1.08, 7.46), *p* = 0.035). The expression of STAT3 is not related to gender (OR = 0.88, 95% CI (0.54, 1.44), *p* = 0.609), age (OR = 0.54, 95% CI (0.21, 1.36), *p* = 0.191), capsular invasion (OR = 2.98, 95% CI (0.23, 38.29), *p* = 0.403), or tumor multiplicity (OR = 0.25, 95% CI (0.003, 19.28), *p* = 0.533) (Table 2).

### 3.3. Subgroup Analysis

**Figure 3:**
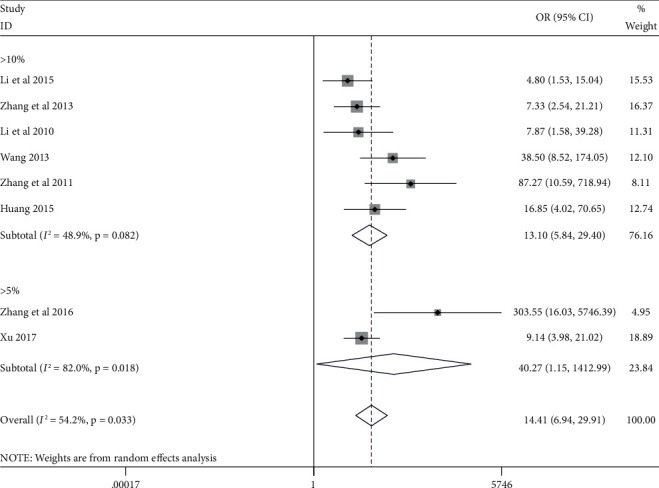
Forest plot for subgroup analysis.

We performed a subgroup analysis based on the cutoff value (>10% vs. > 5%). When the cutoff value is greater than 10%, the expression of STAT3 in thyroid cancer tissue is more highly expressed than normal tissue (OR = 13.10, 95% CI (5.85, 29.40), *p* < 0.0001). At the same time, when the cutoff value is set to >5%, compared with normal tissue, the expression of STAT3 in thyroid cancer tissue is also highly expressed (OR = 40.27, 95% CI (1.15, 1412.99), *p* = 0.042) (Figure 3).

### 3.4. Publication Bias

**Figure 4:**
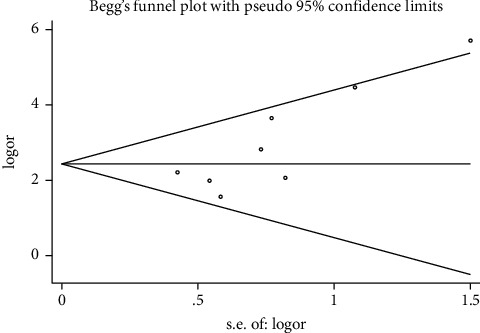
Funnel plot in the meta-analysis of relationship of STAT3 protein expression and thyroid cancer.

For the analysis of STAT3 expression in thyroid cancer tissue and normal tissue, we conducted a sensitivity analysis and publication bias detection. The results suggested no publication bias (Begg's test: *p* = 0.260, Egger's test: *p* = 0.532), as shown in Figure 4.

### 3.5. Sensitivity Analysis

**Figure 5:**
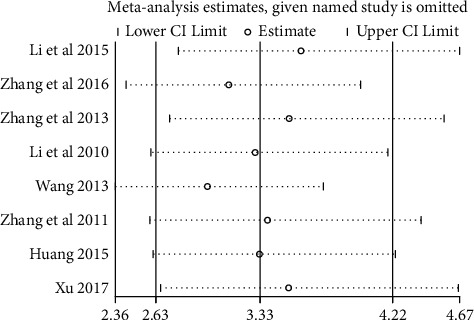
Sensitivity analysis on the relationship of STAT3 protein expression and thyroid cancer.

Results from the sensitivity analysis, shown in Figure 5, suggested that no single study had a significant effect on the overall results, indicating that meta-analysis results are stable.

## 4. Discussion

Currently, the etiology and pathogenesis of thyroid cancer are not clear, and there is a lack of effective treatment methods. Therefore, it is extremely important to explore the occurrence and development of thyroid cancer by searching for oncogenes related to thyroid cancer. In this study, we used a meta-analysis to systematically evaluate the relationship between STAT3 protein expression and the susceptibility and clinicopathological characteristics of thyroid cancer. Results indicated that high expression levels of STAT3 protein increase the risk of thyroid cancer. Therefore, we hypothesize that STAT3 may be one of the oncogenes of thyroid cancer.

As an oncogene, STAT3 plays a vital role in the development of several forms of cancer. In many human cancers, STAT3 is continuously activated, and the phosphorylation of specific tyrosine residue is an important step in STAT3 activation. Once activated, p-STAT3 induces the expression of a variety of genes involved in cell survival, proliferation, migration, invasion, angiogenesis, and immune escape, thus promoting carcinogenic transformation. By inhibiting STAT3 phosphorylation and p-STAT3-mediated DNA transcription and replication, it can promote tumor cell apoptosis [24]. However, established opinions on the tumor-promoting effects of STAT3 have been frequently challenged. Banerjee et al. [25] found that STAT3 has a dual role in breast cancer since STAT3 promotes breast cancer under certain conditions, but it may also be a negative regulator of certain types of breast cancer.

Grabner et al. [26] confirmed that STAT3 has an antitumor effect in mice with lung cancer. The study of STAT3-promoting thyroid carcinogenesis poses similar challenges. The study of Couto et al. [27] revealed that STAT3 is an inhibitor of thyroid tumor growth in preclinical models. Besides, the study of Kim et al. [28] concluded that the activity of STAT3 in papillary thyroid carcinoma tissue is reduced. However, it remains to be explored whether the STAT3 negative regulation of thyroid cancer applies to all histological subtypes, thyroid cancer staging, and distant metastasis progression. Contrary to the research of Couto [27] and Kim et al. [28], we ascertained that STAT3 is highly expressed in thyroid cancer tissue. Also, STAT3 expression increases in thyroid cancer tissue with the metastasis of thyroid cancer lymph nodes or an increase in TNM staging, which subsequently promotes the occurrence and development of thyroid cancer.

Since most patients with thyroid cancer have a good prognosis after surgery, the recurrence rate is low. Even if cancer recurs, the mortality rate remains low. Thus, it is difficult to evaluate the prognosis of patients by survival time only. Therefore, many scholars use clinicopathological characteristics as prognostic indicators, such as gender, age, tumor size, lymph node metastasis, distant metastasis, tissue subtype, and clinical stage [29–34]. This meta-analysis demonstrated that STAT3 is related to the diameter of thyroid cancer tumors, TNM staging, and lymph node metastasis. As a result, the expression of STAT3 protein can be used as an indicator of the prognosis of patients with thyroid cancer.

This study has some limitations. First, the literature included in the meta-analysis lacks survival analysis data. Thus, it is impossible to further evaluate the influence of STAT3 protein on the prognosis of patients with thyroid cancer. Consequently, we can only explain the effect of STAT3 protein expression on survival from pathological characteristics. Because the treatment methods for patients in this study varied, there may be a certain impact on the results. None of the patients included in the meta-analysis received any treatment before surgery, and all patients were treated with surgical resection. Besides, the patients included in this study are only from Asia. The level of medical development in different regions may also affect the results, and different experimental methods may have been used to detect the expression of STAT3.

In summary, our meta-analysis systematically analyzed the relationship between STAT3 protein and thyroid cancer susceptibility and clinical progression. Based on the collected data, we established that STAT3 protein expression is strongly related to thyroid cancer susceptibility, lymph node metastasis, tumor size, and TNM stage. Thus, the expression of STAT3 protein has reference significance for the early diagnosis, treatment, and prevention of thyroid cancer. However, considering the limitations of this study, we require larger study populations, clinical information, multicenter design, and high-quality studies to verify these findings.

## Figures and Tables

**Table 1:** Characteristics and quality score of the studies.

Study	Country	Ethnicity	Detection method	Cutoff value (%)	Antibody supplier	STAT3 (±)		Quality
						Case	Control	
Li et al., 2015	China	Asian	IHC	>10	Abgent, San Diego, CA, USA	32/10	8/12	7
Zhang et al., 2016	China	Asian	IHC	>5	Santa Cruz Biotechnology, Inc	31/0	5/26	7
Zhang et al., 2013	China	Asian	IHC	>10	Santa Cruz Biotechnology, Inc	44/12	8/16	7
Li et al., 2010	China	Asian	IHC	>10	BOSTER Biological Technology Co., Ltd.	23/19	2/13	6
Wang, 2013	China	Asian	IHC	>10	Santa Cruz Biotechnology, Inc	66/24	2/28	7
Zhang et al., 2011	China	Asian	IHC	>10	NA	40/1	11/24	6
Huang, 2015	China	Asian	IHC	>10	NA	73/13	3/9	7
Xu, 2017	China	Asian	IHC	>5	BOSTER Biological Technology Co., Ltd.	47/13	17/43	7

NA, data not available.

**Table 2:** Association between STAT3 expression and clinical features of thyroid cancer patients in meta-analysis.

Factors	Studies (*n*)	Analytical model	OR (95%CI)	*P* value	Heterogeneity	
					*I* ^2^	*P*_h_
Age (<45 vs ≥ 45)	4	REM	0.54 (0.21, 1.36)	0.191	64.00	0.039
Sex (male vs female)	4	FEM	0.88 (0.54, 1.44)	0.609	0	0.898
Tumor size (≤1 cm vs. > 1 cm)	2	FEM	**0.13 (0.05, 0.33)**	**<0.001**	0	0.743
Tumor metastasis (yes vs. no)	6	REM	**2.83 (1.08, 7.46)**	**0.035**	80.70	<0.001
Capsular invasion (yes vs. no)	2	REM	2.98 (0.23, 38.29)	0.403	89.60	0.002
TNM stage (I-II vs. III-IV)	3	REM	**0.40 (0.24, 0.67)**	**<0.001**	82.10	0.004
Tumor multiplicity (single vs. multiplicity)	2	REM	0.25 (0.003, 19.28)	0.533	86.00	0.008

*P* values were obtained by using the “METAN” program in Stata 14.0; *P* value 0.05 was considered as statistically significant. FEM, fixed-effects model; REM, random-effects model; TNM, tumor node metastasis; OR, odds ratio; CI, confidence interval. Bold fonts indicate statistical differences.

## Data Availability

The data used to support the findings of this study are available from the corresponding author upon request.
